# Expression of the inactivating deiodinase, Deiodinase 3, in the pre-metamorphic tadpole retina

**DOI:** 10.1371/journal.pone.0195374

**Published:** 2018-04-11

**Authors:** Karine Le Blay, Laëtitia Préau, Ghislaine Morvan-Dubois, Barbara Demeneix

**Affiliations:** 1 Département Adaptation du Vivant, UMR CNRS, Evolution des Régulations Endocriniennes, Muséum National d’Histoire Naturelle, Paris, France; 2 Zoologisches Institut, Zell-und Entwicklungsbiologie, Karlsruhe Institute of Technology (KIT), Karlsruhe, Germany; National Eye Institute, UNITED STATES

## Abstract

Thyroid hormone (TH) orchestrates amphibian metamorphosis. Thus, this developmental phase is often used to study TH-dependent responses in specific tissues. However, TH signaling appears early in development raising the question of the control of TH availability in specific cell types prior to metamorphosis. TH availability is under strict temporal and tissue-specific control by deiodinases. We examined the expression of the TH-inactivating enzyme, deiodinase type 3 (D3), during early retinal development. To this end we created a *Xenopus laevis* transgenic line expressing GFP from the Xenopus *dio3* promoter region (p*dio3*) and followed p*dio3*–GFP expression in pre-metamorphic tadpoles. To validate retinal GFP expression in the transgenic line as a function of *dio3* promoter activity, we used *in situ* hybridization to compare endogenous *dio3* expression to reporter-driven GFP activity. Retinal expression of *dio3* increased during pre-metamorphosis through stages NF41, 45 and 48. Both sets of results show *dio3* to have cell-specific, dynamic expression in the pre-metamorphic retina. At stage NF48, *dio3* expression co-localised with markers for photoreceptors, rods, Opsin-S cones and bipolar neurons. In contrast, in post-metamorphic juveniles *dio3* expression was reduced and spatially confined to certain photoreceptors and amacrine cells. We compared *dio3* expression at stages NF41 and NF48 with TH-dependent transcriptional responses using another transgenic reporter line: *THbZIP*-GFP and by analyzing the expression of T_3_-regulated genes in distinct TH availability contexts. At stage NF48, the majority of retinal cells expressing *dio3* were negative for T_3_ signaling. Notably, most ganglion cells were virtually both *dio3-*free and T_3_-responsive. The results show that *dio3* can reduce TH availability at the cellular scale. Further, a reduction in *dio3* expression can trigger fine-tuned T_3_ action in cell-type specific maturation at the right time, as exemplified here in photoreceptor survival in the pre-metamorphic retina.

## Introduction

Tissue specific availability of the two main forms of thyroid hormone, thyroxine (T4) and the most biologically active form, triiodothyronine (T_3_), orchestrates the complex developmental program of amphibian metamorphosis. As in other vertebrates, development of the central nervous system is highly T_3_-dependent [1]. This is the case for retinal development, [2, 3]. TH receptors (TR), TRa and TRb, have been detected in the retina of mice, chicken and the African clawed frog *Xenopus laevis* [4, 5]. We previously showed that disrupting *thrb* receptor during development impairs Xenopus retinal formation [4]. More specifically, inducing a mutation in *Xenopus laevis thrb* ligand binding domain causes defects in eye development in embryos [4]. These data suggested that TH could have tissue specific responses during early stages of eye development. However, nothing is known on the cell specific role of TH in amphibian eye development at pre-metamorphic stages and whether certain cell types would be particularly sensitive to excess T_3_.

Thyroid hormone availability in specific tissues is determined by reciprocal activity of activating and inactivating deiodinases. The main activating deiodinase is deiodinase 2 (D2), whereas deiodinase 3 (D3, encoded by *dio3*) is the principal inactivating enzyme in tissue. Deiodinases are essential regulators of TH levels in target cells, notably in the nervous system [6, 7, 8]. The central role of deiodinases in controlling TH availability is exemplified by control of retinal development in multiple vertebrate species [5, 9, 10, 11]. In particular, fine regulation of TH availability by activating and inactivating deiodinases ensures survival and maturation of cone photoreceptors in mice [12]. Deiodinase knockdown in zebrafish eye affects eye size, retinal lamination and strongly reduces the number of rods and cone cells [5]. This result is consistent with the lamination defect observed when impairing deiodinase activity with iopanoic acid in earlier pre-metamorphic Xenopus embryos [4]. When TH deiodination is impaired by iopanoic acid treatment, T_3_ treatment further enhances the deleterious eye phenotype, revealing a crucial role for *thra* as aporeceptor and pinpointing that TH availability is tightly controlled [4] during eye development. D3 plays an essential protective role in inhibiting TH-induced proliferation in CMZ, resulting in an asymmetric growth in *Xenopus laevis* retina during metamorphosis [13, 11], or in protecting certain photoreceptors from excessive T_3_ signaling in the mouse and zebrafish retina [5, 12, 14]. These findings favor the hypothesis of a control of local TH availability mostly by the inactivating deiodinase D3.

The second question raised by this data set is the scale at which local TH availability is controlled, and more precisely, if the control occurs at the tissue level or cellular level. TH roles in proliferation and in the differentiation of cone photoreceptors are well known, but little is known about the protective role of D3 during retinal neurogenesis.

Furthermore, previous studies in *Xenopus* retina addressed the question of the regulation of the stem cells and progenitor cells proliferation [15] or the genesis of different cell types [16, 17, 18, 19] in early development. Other studies addressed the determination of cone and rod photoreceptors during embryonic stages NF33-41 [20, 21]. These studies raised the importance of local environment and extrinsic factors but did not address the role of TH.

As to other species, the role of TH on retinal development has been addressed during embryogenesis in rodents [10, 14], during pro-metamorphosis in fish [5] as well as during metamorphosis in *Xenopus* [4, 11].

As to deiodinase expression, different studies have shown the general pattern expression of *dio3* during embryonic chicken brain development [3, 12] and increasing mRNA levels during zebrafish embryonic development [5, 9] and in brain between stages NF45 and NF48 in *Xenopus* tadpoles [8]. But, no studies have addressed the control of TH availability by D3 on retinal neurogenesis in *Xenopus* pre-metamorphic tadpoles.

To address the question of TH availability in specific retinal cell types, we used a number of complementary experimental approaches. First, we compared expression of *dio3* using both *in situ* hybridization (ISH) and exploiting a p*dio3*-GFP construct, where GFP expression is controlled by part of the Xenopus *dio3* promoter. Second, we compared *dio3* ISH expression with immunohistochemistry for specific retinal cell types. Third, we determined which of these cells types responded to T_3_ signaling by using a well-established *TH/bZIP*-GFP reporter system [22, 23, 24]. Taken together, the results show that *dio3* expression could limit T_3_-dependent responses in specific retinal cells at specific developmental stages.

## Material and methods

### 1. Transgenic and wild type animals

*X*. *laevis* tadpoles and juveniles were obtained by in-house breeding. To obtain eggs, adult frogs were injected with human chorionic gonadotropin (Chorulon) (400 U/female and 200 U/male). Some adult frogs were bought to the CRB (Xenope Biology Resources Centre, Centre de Ressource Biologie Xénope), France (University of Rennes1; http://xenopus.univ-rennes1.fr/).

Animals were reared under a 12-hour light/12-hour dark cycle at 22–23°C. Animals were staged according to Nieuwkoop and Faber (NF) (1956). Juveniles were used when they had developed beyond NF66 and weighed between 1 and 2 g.

To determine T_3_ responsiveness in the retina, we used the *X*. *laevis* transgenic *TH/bZIP* reporter line [22, 23, 24]. The transgenic *TH/bZIP* reporter line contains a series of T_3_ sensitive response elements upstream of the GFP coding sequence providing a direct T_3_ readout. F1 founders were crossed together to produce F2 homozygous transgenic tadpoles.

All animal studies were carried out in accordance with the European Union regulations concerning the protection of experimental animals and approved by the Museum National d’Histoire Naturelle Animal Care and Use Committee, Paris, France. All procedures were approved by the institutional Ethics Committee (Animal Housing Agreement Number: C75-05-01-2, Committee Approval 68.031).

### 2. Generation of the *Xenopus laevis deiodinase 3*–GFP transgenic line

The *X*. *laevis* transgenic line *deiodinase 3*-GFP (p*dio3*-GFP) reporter was obtained by sperm nuclei-mediated REMI method transgenesis [25] using a *dio3* promoter driven-GFP expressing construct.

Injections were carried out so that the transgene was inserted into the genome prior to the first cleavage. The DNA plasmid construct used for this reporter line corresponds to a clone representing a part of the *Xenopus tropicalis dio3* promoter locus and upstream regulatory elements. The DNA clone represents 1448 pb of the full *Xt dio3* 4915 pb promoter locus. The cDNA clone was from 454 pb of the 3’end *Xt dio3* promoter locus. The PCR product was initially obtained with the ExTaq TaKaRa kit (Ozyme, ref: TAKRR013A) from *X*. *tropicalis* tadpole tail genomic DNA extract.

PCR primers were designed against the promoter *dio3* locus. Forward and reverse primers were 5’CGGGGAAGATATGTGAAGGA3’ and 5’GGGCTCCCAGGATGATCTGA 3’, respectively. The latest *Xenopus tropicalis* genome annotation, confirms the position of the clone in the promoter. This PCR product was sub-cloned into a pGlow-GFP vector (Invitrogen pGlowTOPO cloning kit ref: 45–0021) and verified by sequencing.

F0 or F1 transgenic founders were crossed with wild type adult to produce F1 or F2 transgenic tadpoles that were used for the characterization experiments.

### 3. Tadpole treatment

The *X*. *laevis* transgenic (p*dio3*-GFP) reporter tadpoles NF48 were treated for 24 h, with 10^−8^ M T_3_ (Sigma, St Quentin Fallavier, France), with 5.10^-6^M IOP (TCI Europe, Zwijndrecht, Belgium) or with 10^-8^M T_3_ + 5.10^-6^M IOP (diluted in 0.1% ethanol) [4]. Vehicle control group is used in 0.1% ethanol. The experiment was performed twice, with 5–8 animals per group.

### 4. Sample collection and preparation

Tadpoles and juveniles of both transgenic reporter lines were deeply anesthetized by submersion in 0.1% tricaïne methanesulfonate anesthesia (MS-222, Sigma-Aldrich) and euthanized by decapitation. Whole heads of NF48 tadpoles and juveniles were fixed overnight at 4°C in 4% paraformaldehyde (PFA, Sigma-Aldrich, Saint Quentin Fallavier, France) in phosphate buffered saline (PBS pH 7.4). Samples were briefly washed three times (15 min.) in PBS and placed overnight in 15% sucrose PBS. Samples were embedded in Tissue-Tek and stored at -80°C. Coronal cryosections (16 micrometers) were performed on whole heads and analyzed on eyes. Sections were stored at -80°C prior to immunohistochemistry or *in situ* hybridization.

### 5. Immunohistochemistry for GFP and Opsin Blue, GFP and Rhodopsin, GFP and ChX10

Coronal whole heads cryosections were briefly rehydrated with phosphate buffer salt PBS (pH 7.4) and Tween 20 (0.1%), then post-fixed with 4% PFA for 10 min. Sections were washed in PBS-Tween 20 (0.1%) and blocked with 10% normal goat serum in PBS-Tween 20 (0.1%) (both from Sigma-Aldrich) to reduce non-specific binding. Sections were then incubated overnight at 4°C with primary antibody chicken polyclonal GFP (Abcam, Ab 13970) or rabbit polyclonal GFP (Invitrogen, ref: 11122) diluted 1:300 in PBS-Tween 20 (0.1%) containing 5% normal goat serum (NGS), according to the species in which the second primary antibody was generated. The slides were rinsed with fresh PBS-Tween 20 (0.1%) and incubated two hours at room temperature (RT) with secondary antibody (Alexa fluor 488 goat anti-rabbit, Invitrogen A-11034 Carlsbad, CA, USA) diluted 1:500 in PBS-5% normal goat serum-0.1% Tween 20. Then, sections were incubated with the second primary rabbit polyclonal antibody either Opsin Blue, a marker of Opsin S (Millipore AB 5407) diluted 1:250 or mouse monoclonal Rhodopsin (Sigma-Aldrich R 5403) diluted 1:500 or sheep polyclonal ChX10 (Exalpha X1180P) diluted 1:200 in PBS-Tween 20 (0.1%) containing 5% normal goat serum. Slides were rinsed with fresh PBS-Tween 20 (0.1%) and incubated two hours at RT with secondary antibody Alexa fluor 568 goat anti-rabbit, (Invitrogen A-11036) for Opsin-Blue or Alexa fluor 594 donkey anti-mouse, (Invitrogen A-21203) for Rhodopsin or Alexa 594 donkey anti-sheep IgG (Invitrogen A-11016) for ChX10 diluted 1:500 in PBS-Tween 20 (0.1%) with 5% NGS. Immunostained retinas were rinsed several times in PBS-Tween 20 (0.1%) and mounted with Prolong Gold antifade reagent with DAPI (Invitrogen P-36931) and a cover slip.

### 6. Immunohistochemistry for GFP and GABA or GFP and Parvalbumin

Coronal cryosections were briefly rehydrated with phosphate buffer salt PBS (pH 7.4) and Triton X-100 (0.3%). Sections were blocked with 10% normal goat serum in PBS-Triton X-100 (0.3%) (both from Sigma-Aldrich) to reduce non-specific binding during 2 hours at room temperature. Sections were then incubated overnight at 4°C with primary antibody chicken polyclonal GFP (Abcam, Ab 13970) diluted 1:300 in PBS-Triton X-100 (0.3%) containing 5% normal goat serum (NGS). The slides were rinsed with fresh PBS-Tween 20 (0.1%) and incubated two hours at room temperature (RT) with secondary antibody (Alexa fluor 488 goat anti-chicken, Invitrogen A-11039 Carlsbad, CA, USA) diluted 1:500 in PBS-5% normal goat serum-0.3% Triton X-100. Then, sections were incubated with the second primary rabbit polyclonal antibody GABA, gamma-Aminobutyric Acid (Immunostar 20094) diluted 1:1500 or mouse monoclonal Parvalbumin IgG1 (Millipore MAB1572) diluted 1:100 in PBS-Triton X-100 (0.3%) containing 5% normal goat serum. Slides were rinsed with fresh PBS-Tween 20 (0.1%) and incubated two hours at RT with secondary antibody Alexa fluor 647 goat anti-rabbit, (Invitrogen A-21245) for GABA or Alexa fluor 568 goat anti-mouse IgG1, (Invitrogen A-21124) for Parvalbumin diluted 1:500 in PBS-Triton X-100 (0.3%) with 5% NGS. Immunostained retinas were rinsed several times in PBS-Tween 20 (0.1%) and stained by DAPI (Sigma-Aldrich) for 7 min, rinsed several times in PBS-Tween 20 (0.1%), another time and mounted with Prolong Gold antifade reagent (Invitrogen P-36930) and a cover slip.

The immunostained retinas were visualized with a LEICA DM 5500 B microscope equipped with a LEICA CTR 5500 lens and for epifluorescence with a PRIOR Lumen 200 system.

Fluorescent image acquisitions were carried out using a ZEISS LSM 710 system confocal microscope by channels or spectral mode acquisitions and the Zen 2011 software acquisition at the J. Monod Institute (ImagoSeine platform, Jussieu University, Paris, France).

### 7. *In situ* Hybidization (ISH)

Probes for *dio3* cDNA were isolated by RT-PCR using a pool of RNA extracted from embryos and tadpoles at several stages.

The PCR fragment for p*dio3* (296 bp, forward: TCGGTGCACAATAGTCGGG and reverse:CTTCTGCCCGTGCCACAC) were cloned using the TOPO TA cloning kit dual promoter (Invitrogen, Carlsbad, CA, USA) and sequenced to check orientation. The *dio3* mRNA probe was synthesized using T7 enzyme 25 (Roche, Basel, Switzerland) and labeled with digoxigenin (Roche, Basel, Switzerland). ISH was performed on cryosections. Slides were briefly rehydrated with PBS (pH 7.4) then post-fixed with 4% PFA during 10 min. Then slides were washed with PBS Tween 20 (0.1%). Tissues were permeated with proteinase k (5.10^−6^ g/ml, Sigma, ref. P4850) for two minutes. Slides were then post-fixed with 4% PFA during 10 min follow by wash with PBS Tween 20 (0.1%). To prevent RNA interaction with protein, slides were incubated with 100 mM Triethanolamine/0.25% anhydride acid acetic during 10 min. Slides were washed with PBS Tween 20 (0.1%) and then pre-hydrated with hybridization buffer (formamide 50%, SSC 5X, ARNt grade VI à 1 mg/ml, heparin 100.10^−6^ g/ml, Denhart’s 1X, Tween 20 (0.1%), CHAPS 10 mM 0.1%, EDTA 10 mM) during 1 hour. After denaturation *dio3* probe was used at 1 ng/μl concentration and incubated on slides overnight at 60°C. Slides were washed several times with SSC buffer (saline sodium chloride citrate Sigma, ref.93017) at 65°C and then blocked with 10% NGS conjugated to alkaline phosphatase (1/2000, Roche) in 5% NGS/PBS/Tween 20 (0.1%). Slides were washed several times in maleic acid buffer. Signals were revealed using BM purple (Roche, ref.11442074001). Reactions were stopped by washes with PBS Tween 20 (0.1%). Slides were mounted with Prolong (Invitrogen, Carlsbad, CA, USA).

ISH on retina sections was visualized using a LEICA DM 5500 B microscope equipped for visible light with LEICA CTR 5500. Image acquisition exploited a DFC 450 C Camera and the Leica Application suite LAS version 4.1.0 software acquisition.

### 8. RNA isolation and reverse transcription (RT)

RNA extraction was performed by RNA isolation from micro-scale, using RNAqueous Micro kit (Invitrogen AM 1931). For mRNA extraction from eyes, two steel balls were used for each sample in 100 microliters of lysis solution. Eyes were homogenized by using a tissue lyser at 30 Hz twice during 1 min. Ethanol 100% (50 microliters) was added and the lysate was placed on a micro-filter cartridge before centrifugation (16,400 x g, 20 sec.). The cartridge was rinsed three times with a wash solution before RNA elution. A warm (75°C) RNA elution solution (18 microliters) was used before centrifugation (twice 16,400 x g, 30 sec. at room temperature). A DNAse was used (22 min. at 37°C) and an inactivation reagent was added before centrifugation (16,400 x g, 2 min.). Concentrations of RNA were determined by using a NanoDrop (ThermoScientific, Rockford, IL). RNAs were stored in TRIS 10 mM /EDTA 0.1 mM (PH 7.4) at -80°C.

Extracted total RNA (300 ng) was used for reverse transcription (RT) using a High Capacity cDNA RT kit (Applied Biosystems, Foster City, CA) with the addition of RNase inhibitor. A control for genomic DNA (RT- reaction: all reagents and RNA except reverse transcriptase) was performed for each group (Ctrl, T_3_, IOP, IOP+T_3_).

### 9. Real-time PCR quantification

The amount of each RNA transcript was estimated by relative quantitative real-time PCR (qRT-PCR) using Power SYBR green master mix and a Quant Studio Flex 6 (Applied Biosystems). 5 to 8 biological replicates were performed for each group. A 1:10 dilution of each cDNA was run in triplicate on a 384-well plate for each primer pair (Table 1) (intra assay variability) by using thermal cycling parameters: 95°C for 10 min, 95°C for 15 sec and 60°C for 1 min (40 cycles) and an additional step for dissociation curves was performed for all plates. Results were normalized with the expression of reference gene *odc*. DDCT method was used to estimate fold change of expression when compared to the untreated (CTL) group. PCR primer sequences are designed previously [26] for the housekeeping gene *odc*. Primers list is provided in Table 1.

**Table 1:** Primers list.

Primer sequences		
Gene	Forward primer	Reverse primer
***eGFP***	5’ ACA GGA TGA GGA TCG TTT CG 3’	5’ TGT CTG TTG TGC CCA GTC AT 3’
***dio3***	5’ CAC AAA AAG TGC GAC CAA ACG 3’	5’ GCC TTG TTG CAG TTT ACT 3’
***thrb***	5’ ATA GTT AAT GCG CCC GAG GGT GGA 3’	5’ CTT TTC TAT TCT CTC TCC ACG CTA GC 3’
***thibz***	5’ CCC GTC TCC GTG CTG AAC T 3’	5’ GGT CAC GTA CCA GGC CAA A 3’
***klf9***	5’ TGT GGC AAA GTT TAT GGG AAG TCT 3’	5’ GGC GTT CAC CTG TAT GGA CTC T 3’
***odc***	5’ GCT TCT GGA GCG GGC AAA GGA 3’	5’ CCA AGC TCA GCC CCC ATG TCA 3’

### Statistical analysis

For multiple comparison analysis, Non-parametric ANOVA was performed, followed by a Kruskall Wallis test (PRISM7). Heatmap was performed on Fold Changes (PRISM7), and correlation analysis was performed on XLSTAT.

## Results

### Dynamic expression of retinal *dio3* in the pre-metamorphic *Xenopus laevis* retina

**Fig 1:**
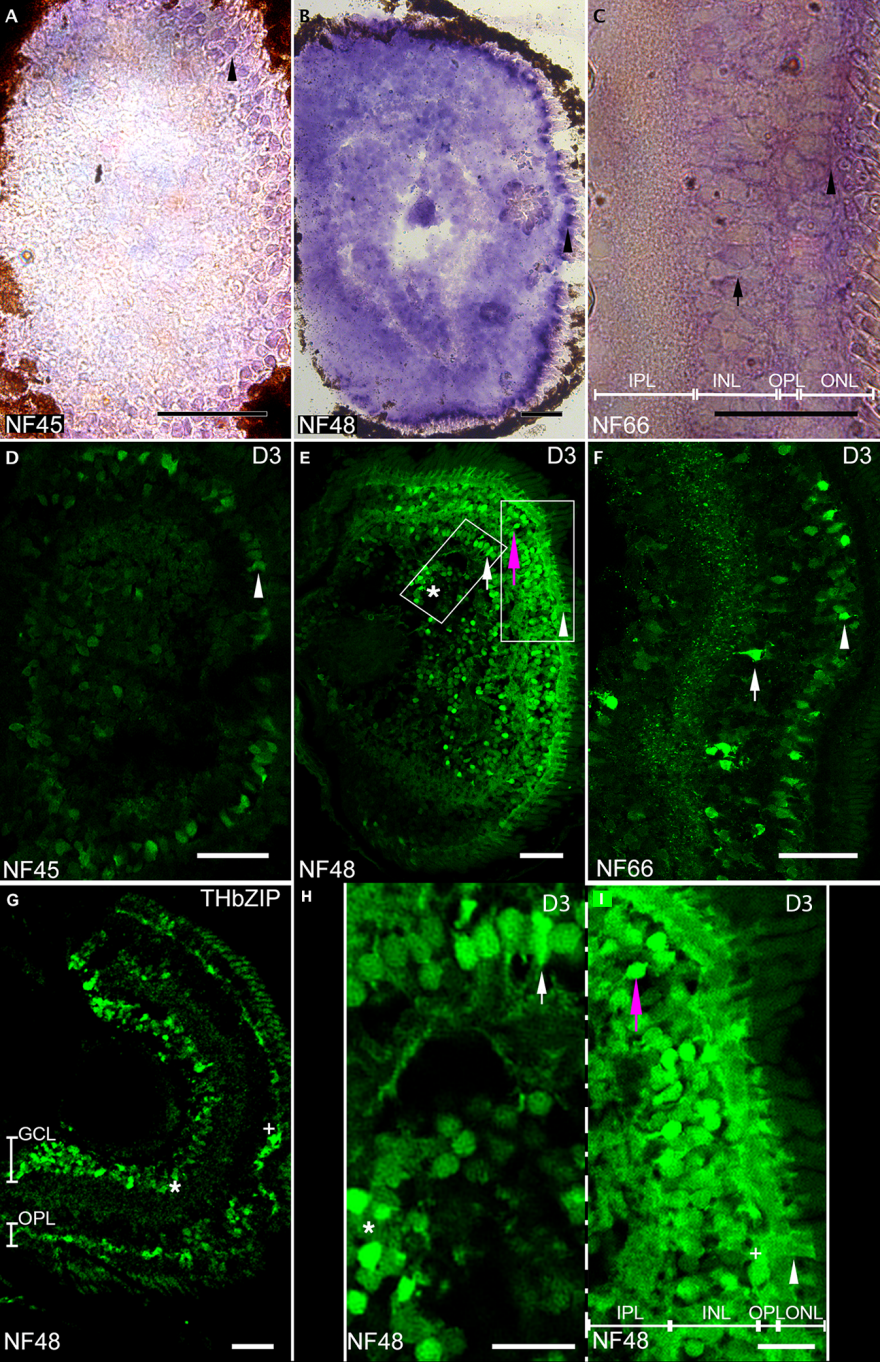
Comparison of GFP expression in transgenic p*dio3* reporter line to endogenous *dio3* expression. Fig 1A–1C: Endogenous *dio3* in situ hybridization on retina coronal sections from animals at NF45 (Fig 1A), NF48 (Fig 1B), NF66 (Fig 1C) stages. Fig 1D–1F: GFP immuno-labeling on retina coronal sections at NF45 (Fig 1D), NF48 (Fig 1E, 1H and 1I), NF66 (Fig 1F) stages. Comparison of GFP expression between tadpoles from the p*dio3* transgenic line (Fig 1E) and from *TH/bZIP* transgenic T_3_ reporter tadpoles at NF48 stage (Fig 1G). Orientation is with lens on the left and retina on the right. Photoreceptor cell bodies (arrow head: Fig 1A–1F and 1I). Amacrine cells (arrow: Fig 1C, 1F, 1E and 1H). Bipolar neurons (magenta arrow: Fig 1E and 1I). Ganglion cells (asterisk: Fig 1E, 1H and 1G). Scale bars: 50 microns.

To address the role of *dio3* in pre-metamorphic retina, we cloned part of the *X*. *tropicalis dio3* promoter and generated a transgenic p*dio3*-GFP reporter line. GFP immunostaining was used to follow p*dio3*-reporter GFP expression. To validate the *dio3* transgenic GFP reporter signal in the retina, we compared it to the endogenous signal obtained with ISH using antisense probes against *dio3* (Fig 1A–1F). Expression of *dio3* (Fig 1A and 1B) and p*dio3-*reporter GFP (Fig 1D, 1E, 1H and 1I) were examined by ISH on head cryo-sections in tadpoles at stages NF45 and NF48 and in the froglet at stage NF66 (Fig 1C and 1F).

At stage NF45, both the *dio3* ISH signal and GFP expression were limited to a very few ganglion cells and in a subset of the photoreceptors of the outer nuclear layer, as judged by cell morphology and anatomical localization (Fig 1A and 1D). At stage NF48, both the *dio3* ISH signal and GFP expression showed higher intensity and a wider distribution, being present in all retinal cell types: ganglion cells, inner nuclear layer cells (bipolar neurons, horizontal neurons and amacrine cells) and in the outer nuclear layer cells (basal part of the photoreceptors) (Fig 1B, 1E, 1H and 1I). Cell morphologies were determined according to Wong et al. [27]. At stage NF66 corresponding to a post-metamorphic juvenile stage, *dio3* expression was more restricted and localized in the outer nuclear layer and in the inner nuclear layer. More specifically, both ISH and GFP immunocytochemistry showed *dio3* expression in certain photoreceptor types and in Amacrine cells (Fig 1C and 1F).

### D3 expression generally corresponds to lack of T_3_ responsiveness

**Fig 2:**
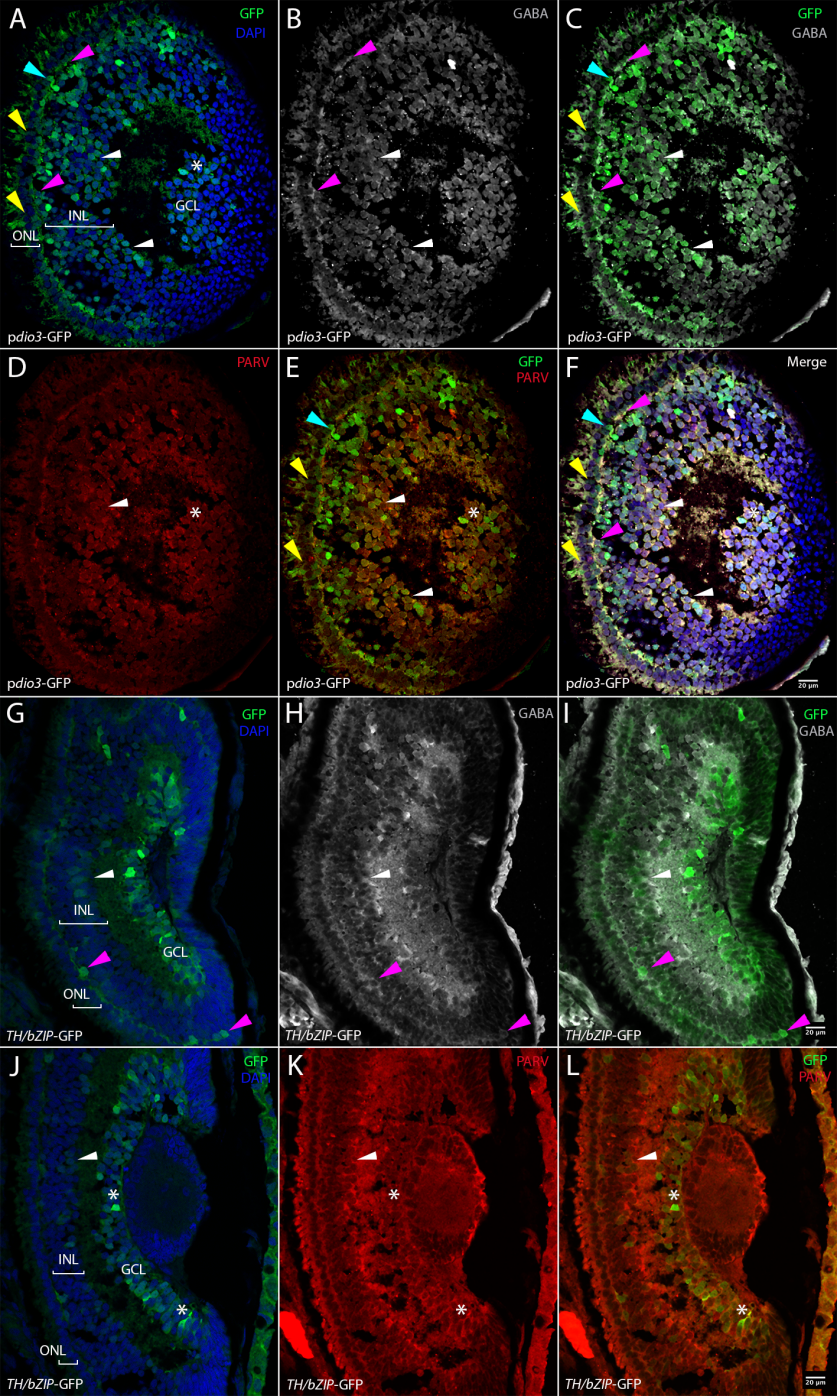
Comparison of p*dio3*-GFP and *TH/bZIP*-GFP expression pattern in NF41-42 reporter tadpoles. Fig 2A–2F: p*dio3*-GFP transgenic reporter tadpoles, Fig 2G–2L: *THbZIP*-GFP (T_3_ sensor) reporter tadpoles. Fig 2A–2I: lateral plane sections. Fig 2J–2L: median plane section. Fig 2A, 2G and 2J: DAPI/GFP co-staining, Fig 2B and 2H: GABA labeling in horizontal neurons (magenta arrow heads) and amacrine cells (white arrow heads). Fig 2C and 2I: GFP/GABA co-labeling in horizontal neurons (magenta arrow heads) and amacrine cells (white arrow heads). Fig 2D and 2K: Parvalbumin PARV labeling in amacrine cells (white arrow head) and ganglion cells (asterisks). Fig 2E and 2L: GFP/PARV co-labeling in amacrine cells (white arrow head) and ganglion cells (asterisks). Fig 2F: merge of all channels shown in Fig 2A–2E. Yellow arrow heads indicate photoreceptors and blue arrow heads indicate the bipolar neurons. Scale bars: 20 microns.

To determine which retinal cells express D3 and those that respond to T_3_, we compared the GFP expression in F2 tadpoles for two transgenic reporter lines, respectively p*dio3*-GFP and *TH/bZIP*-GFP. Retinal GFP immuno-labeling was examined on 16 micrometers coronal whole head cryo-sections at stage NF 41–42 (Fig 2) and NF48 (Fig 1E, 1H, 1I and 1G) (see Materials and Methods).

At stage NF41-42 D3 activity, as judged by GFP expression in p*dio3*-GFP tadpoles, is observed in numerous photoreceptors of the outer nuclear layer (yellow arrow heads, Fig 2A, 2C, 2E and 2F and Table 2) and in some bipolar neurons (blue arrow heads, Fig 2A, 2C, 2E and 2F and Table 2), whereas no T_3_-response is detected in photoreceptors nor in bipolar neurons of the inner nuclear layer (INL) (Fig 2G, 2I, 2J and 2L and Table 2). Furthermore, the photoreceptors that are GFP positive in p*dio3*-GFP tadpoles are in the dorso-central area (Fig 2A).

**Table 2:** A summary of p*dio3*-GFP expression and T_3_—responsiveness.

		*TH/bZIP*-GFP		p*dio3*-GFP	
		NF41	NF48	NF41	NF48
Outer Nuclear Layer	**Photoreceptors**	-	-	+	++
Inner Nuclear Layer	**Bipolar neurons**	-	-	+	+++
	**Amacrine cells**	+/-	-	+	+++
Ganglion Cell Layer	**Retinal ganglion cells**	++	+++	+	+/-
Outer Plexiform Layer	**Horizontal cells dorsal zone**	+	++	++	+++
	**Horizontal cells ventral zone**	++	+++	+	+

The horizontal neurons (magenta arrow heads) of the outer plexiform layer that are GABA positive cells (Fig 2B and 2H) are also GFP positive in p*dio3*-GFP (Fig 2C and 2F and Table 2) and in *TH/bZIP* reporter tadpoles (Fig 2I, Table 2). Similarly, numerous amacrine cells (white arrow heads) in the inner nuclear layer, that are GABA/PARV positive cells (Fig 2B, 2D, 2H and 2K) are GFP positive in p*dio3*-GFP (Fig 2C, 2E and 2F and Table 2) and in *TH/bZIP*-GFP lines (Fig 2I and 2L and Table 2). However, they are less numerous in the *TH/bZIP* reporters. A ganglion cells subset (white asterisk) is Parvalbumine PARV positive (Fig 2D) in the ganglion cell layer and some of them are also GFP positive, in p*dio3*-GFP tadpoles (Fig 2E and 2F and Table 2). In *TH/bZIP*-GFP tadpoles, GFP expression is seen in a large subset of ganglion cells (Fig 2K and 2L and Table 2).

At stage NF48, widespread GFP expression was observed in p*dio3* tadpoles (Fig 1, 1E, 1H and 1I), a fact that was reflected by limited T_3_ responses (lower *TH/bZIP*-driven GFP expression) notably in retinal ganglion cells, noted as “*” in Fig 1 and horizontal neurons (outer plexiform layer), noted as “+” (Fig 1G, Table 2). The p*dio3-*GFP expression displayed a strong dorso-ventral gradient (Fig 1E), which contrasted with the higher levels of *TH/bZIP*-driven GFP in the ventral area, mostly for the outer plexiform layer (Fig 1G). Thus, higher D3 levels appear to restrict T_3_-responsiveness in the dorsal retina at this stage.

Furthermore, at this stage, no *TH/bZIP*-driven GFP signal was seen in any cell type in the inner nuclear layer as in bipolar neurons or amacrine cells (Fig 1G, Table 2), which corresponded to the high expression of p*dio3*-driven GFP at the same stage ((Fig 1E, 1H and 1I); note the magenta arrow indicating a bipolar neuron in Fig 1E and 1I and the white arrow indicating an amacrine cell in Fig 1E and 1H).

Similarly, at stage NF48, the outer plexiform layer was GFP positive in both transgenic lines, reflecting a certain level of T_3_ responsiveness despite discernable D3 activity.

Indeed, some interneurons (horizontal cells, noted as “+”) respond to T_3_ (Fig 1G, Table 2) and yet display limited *dio3* expression (Fig 1E and 1I and Table 2).

At stage NF48, most ganglion cells, (noted as “*”) did not express *dio3* and therefore could respond to T_3_ as confirmed by their positive *TH/bZIP* response (Fig 1G, Table 2). As expected, most photoreceptors express *dio3* (noted as “white head arrow in Fig 1E and 1I) and do not respond to T_3_ (Fig 1G, Table 2). Theses results suggest that *dio3* expression limits T_3_ responsiveness in specific cells too.

### Opsin S-expressing cones, rods and bipolar neurons express p*dio3*-GFP at stage NF48

**Fig 3:**
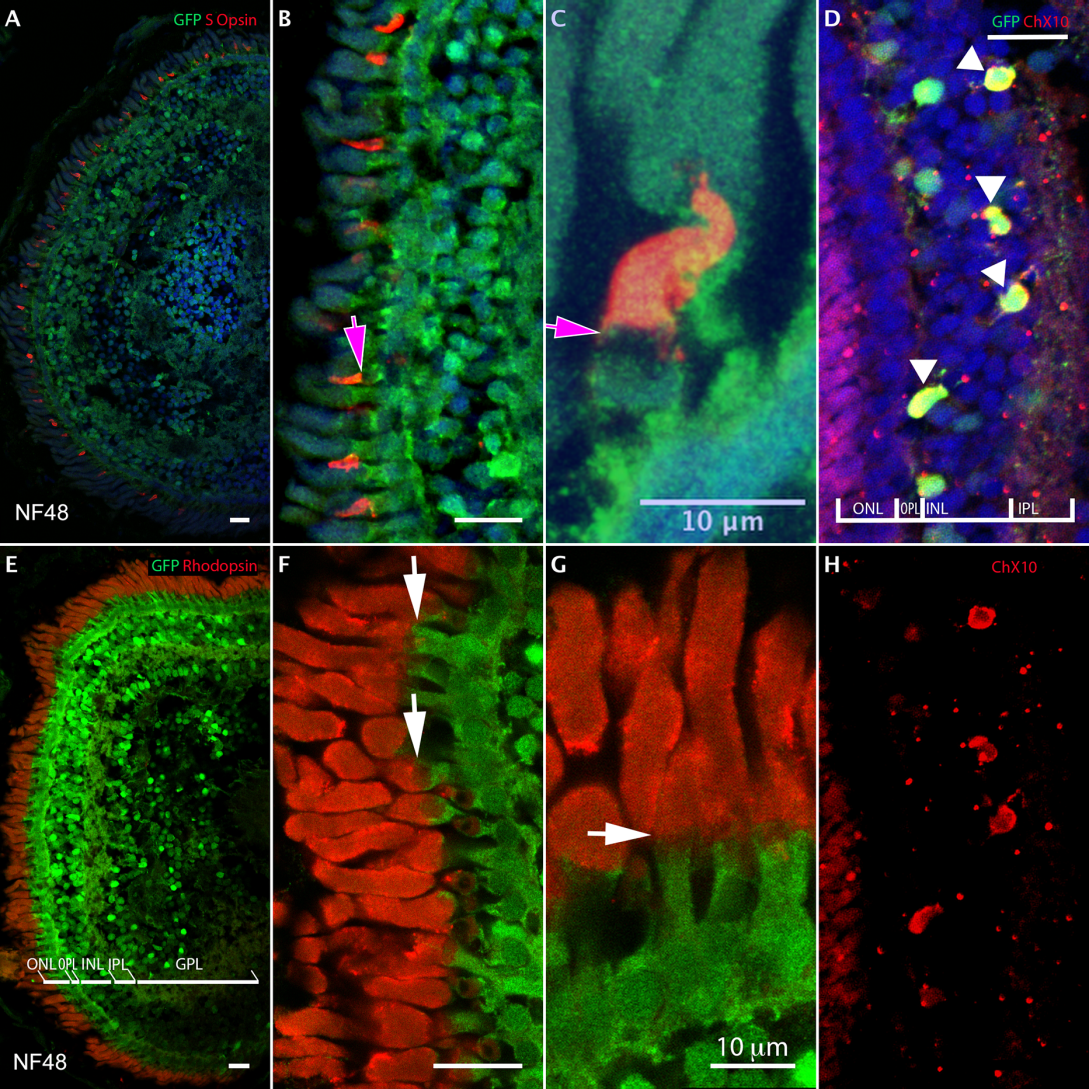
Multiple double-immuno-labeling with GFP (green) of *dio3* transgenic reporter line and Opsin-Blue or Rhodopsin or ChX10 (red) in NF 48 tadpoles. Opsin S cones co-expressing GFP and Opsin-S (Fig 3A–3C; magenta arrow: Opsin S cones; Fig 3B, 3C cone expressing GFP and apical cone expressing Opsin S). Rod cells co-expressing GFP and Rhodopsin (Fig 3E–3G; white arrow: rod Fig 3F and 3G body rod expressing GFP and apical rod expressing Rhodopsin). Bipolar neurons co-expressing GFP and ChX10 (white head arrow: Fig 3D, yellow cells); ChX10 channel from Fig 3D in Fig 3H. Scale bars: 20 microns (Fig 3A, 3B, 3D, 3E, 3F and 3H). Scale bars: 10 microns (Fig 3C and 3G).

To determine more specifically in which retinal cell types p*dio3*-GFP expression was found we used double immunochemistry with established markers of each retinal cell type (see Materials and Methods). At stage NF48, p*dio3*-driven GFP expression was found to co-localize with markers of a large proportion of cone cells expressing Opsin Blue (Opsin S), noted by a magenta arrow in (Fig 3A, 3B and 3C), certain bipolar neurons (ChX10), noted by white head arrows (Fig 3D and 3H) and with all Rhodopsin positive rods, noted by white arrows (Fig 3E–3G).

Thus, as shown in Fig 3A, at stage NF48 GFP expression (green) was found in all retinal cell types in all layers, except in the ganglion cells layer where GFP expression is localized in few ganglion cells. In the outer nuclear layer, it only co-localizes with the body of the Opsin S cones (Fig 3A, in red). Co-localization of p*dio3*-GFP with several Opsin S cone body was observed and shown with magenta arrow in Fig 3B and 3C (zoom) (in red, with magenta arrow).

Another section revealed co-localization of GFP with Rhodopsin (in red), a specific marker of red rods (Fig 3E–3G for different magnifications).

These results suggest that at stage NF48, *dio3* expression limits T_3_ availability, thereby controlling Opsin S cones number and rods survival.

### *dio3* expression prevents induction of T_3_-responsive genes

**Fig 4:**
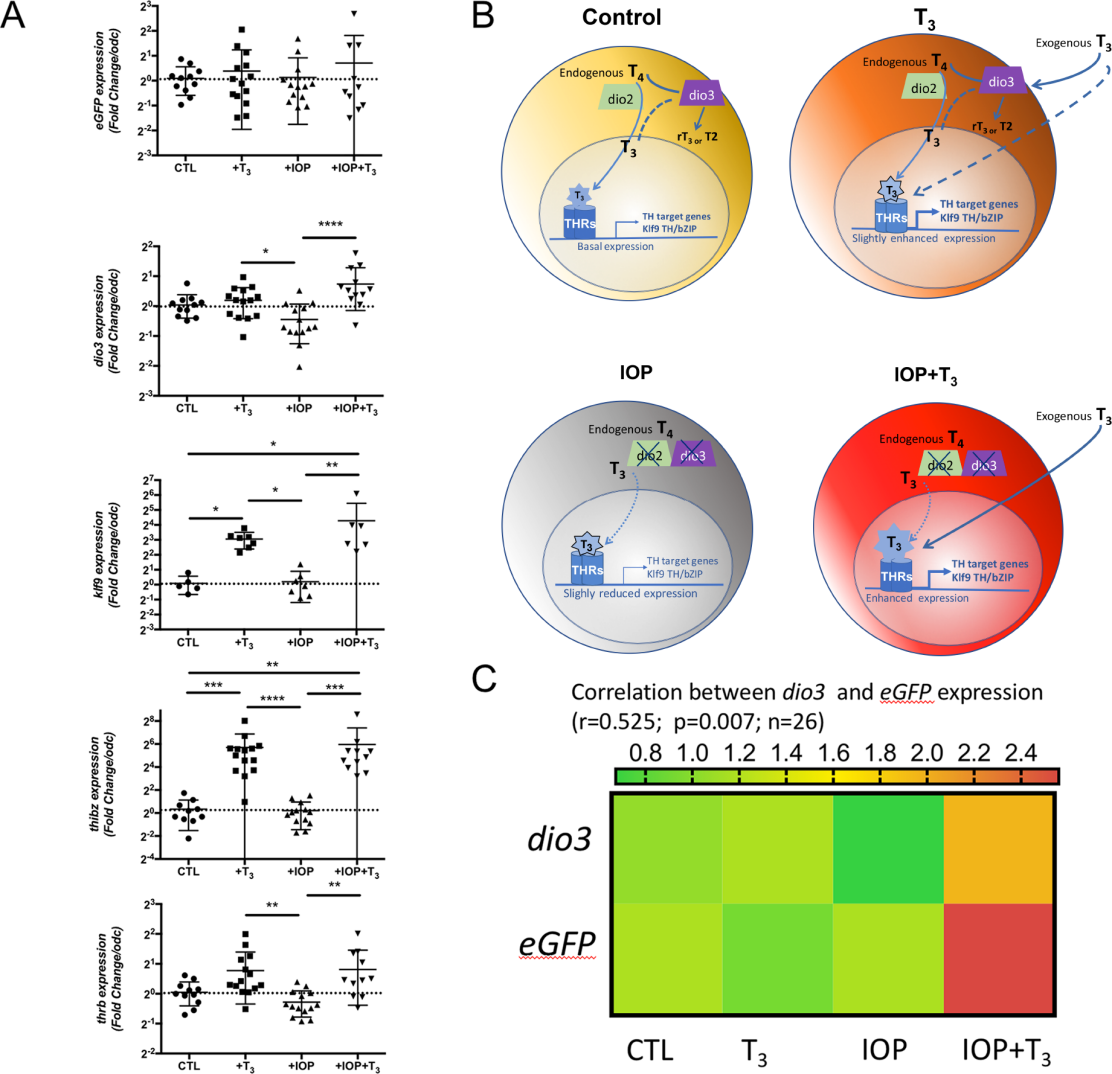
Expression of *dio3* contributes to modulate T_3_ transcriptional response in the developing retina. Fig 4A. Real-time q-PCR analysis of *eGFP*, *dio3*, *klf9*, *thibz* and *thrb* for their T_3_ transcriptional response in NF48 eye from reporter transgenic line p*dio3*-GFP. Gene expression was normalized against *odc*. mRNA levels from vehicle controls (CTL) were used as reference values. Results pooled from two to three independent experiments are represented as scatter dot plots mean with SD. 14≥n≥6 per group. Non-parametric ANOVA, Kruskall Wallis with uncorrected Dunn’s test (PRISM7) was used to assess statistical significance. *, p<0.05, **, p<0.01; ***, p<0.001. Fig 4B. Working model for the local control of T_3_ local availability. Fig 4C. Heatmap of mean expression for each group illustrating the correlation between endogenous *dio3* expression and *eGFP* expression in p*dio3*-GFP tadpole retina.

In order to determine whether *dio3* expression contributes to local control of TH availability in the developing eye, we treated NF48 p*dio3*-reporter tadpoles with T_3_, IOP or IOP+T_3_ (see Materials and Methods for details) and analyzed T_3_ target genes expression (Table 1). A highly significant increase (p<0.001) in *dio3* expression was seen between IOP and IOP+T_3_, but not between CTL and T_3_ treated groups (Fig 4A). This difference in T_3_ responsiveness can be interpreted in the light of the sensitivity of both activating and inactivating deiodinases to IOP [4] (Fig 4B). In the presence of T_3_, despite the presence of IOP, we observed an increased expression of the canonical T_3_-responsive genes *klf9* and *thibz*. Of note, IOP treatment itself, induces no significant change (as compared to controls) in *GFP*, *dio3* and T_3_ target gene expression (Fig 4A). This result indicates that the local T_4_ to T_3_ conversion by D2 is insufficient to trigger T_3_ target gene expression in the retina as a whole. In IOP+T_3_ treated group, *dio3*, *klf9*, *thibz* and *thrb* expression increases when compared to control group, showing that the cells expressing these genes are T_3_-responsive. When comparing *dio3* and *GFP* expression (all observations pooled), a significant correlation (p = 0.007) is seen between *dio3* and *GFP* transcript levels (r = 0,525) (Fig 4C). This observation strengthens the argument that *GFP* can be used as an indicator of *dio3* expression levels.

## Discussion

Several studies have shed light on the role of deiodinases in determining sensory organ development in vertebrates. This is especially the case in the eye, where TH-related development is controlled by the timing of *Dio3* expression in mouse retina [12, 28] and *dio3* in *Zebrafish* eye [5], as well as in *Xenopus* retina during metamorphosis [11].

In mouse retina, *Dio3* is expressed in immature mouse retina. Ng et al. [12] showed that *Dio3*-/- mice lost 80% of cones through neonatal cell death. Their results suggest that *Dio3* expression limits hormonal exposure of the cones ensuring cone survival and opsin (S and M) patterning, required for cone adaptive function during development. In zebrafish, knockdown of *dio3* by morpholinos causes reduced eye size and a strong reduction in rods and all four cones types. This result suggests the importance of *dio3* as a central player for zebrafish eye development. However, the roles of *dio3* on the survival and the patterning of Opsin type photoreceptors have not yet been addressed in *Xenopus* retina development, notably during pre-metamorphic stages. What is more, in Xenopus, there were no data on TH availability nor on the role of deiodinase 3 expression in other retinal cell types.

Our experiments were designed to address these questions: how TH availability is controlled, and more precisely, if the control occurs at the tissue or cellular level and in which cells.

In the mouse retina, TH availability appears to control survival and patterning of specific retinal cells. Notably, a TH gradient is observed that may play a role in establishing the gradient of M-opsin [29]. In contrast, in *Xenopus* retina, there is a spatial gradient of *dio3* during metamorphosis, with a higher level in the dorsal retina [11]. These authors demonstrated that the dorsal ciliary margin zone (CMZ) cells are resistant to exogenous TH at stage 50–54, but they noted that an increase in proliferation of these cells was induced with a low concentration of T_3_ when D3 activity was inhibited. In contrast, D3 overexpression inhibited TH-induced proliferation of the ventral CMZ cells [11]. This localized expression of *dio3* in the dorsal CMZ leads to the asymmetric growth of the frog retina, but the question of the role of *dio3* expression in the eyes of pre-metamorphic tadpoles was not addressed. Interestingly, in pre-metamorphic tadpoles, maximal *dio3* mRNA levels are found in whole tadpoles at NF46-NF48 stages [22], crucial stages before the first pro-metamorphosis stage NF 53, where generalized competence to respond to T_3_ is observed [30].

**Fig 5:**
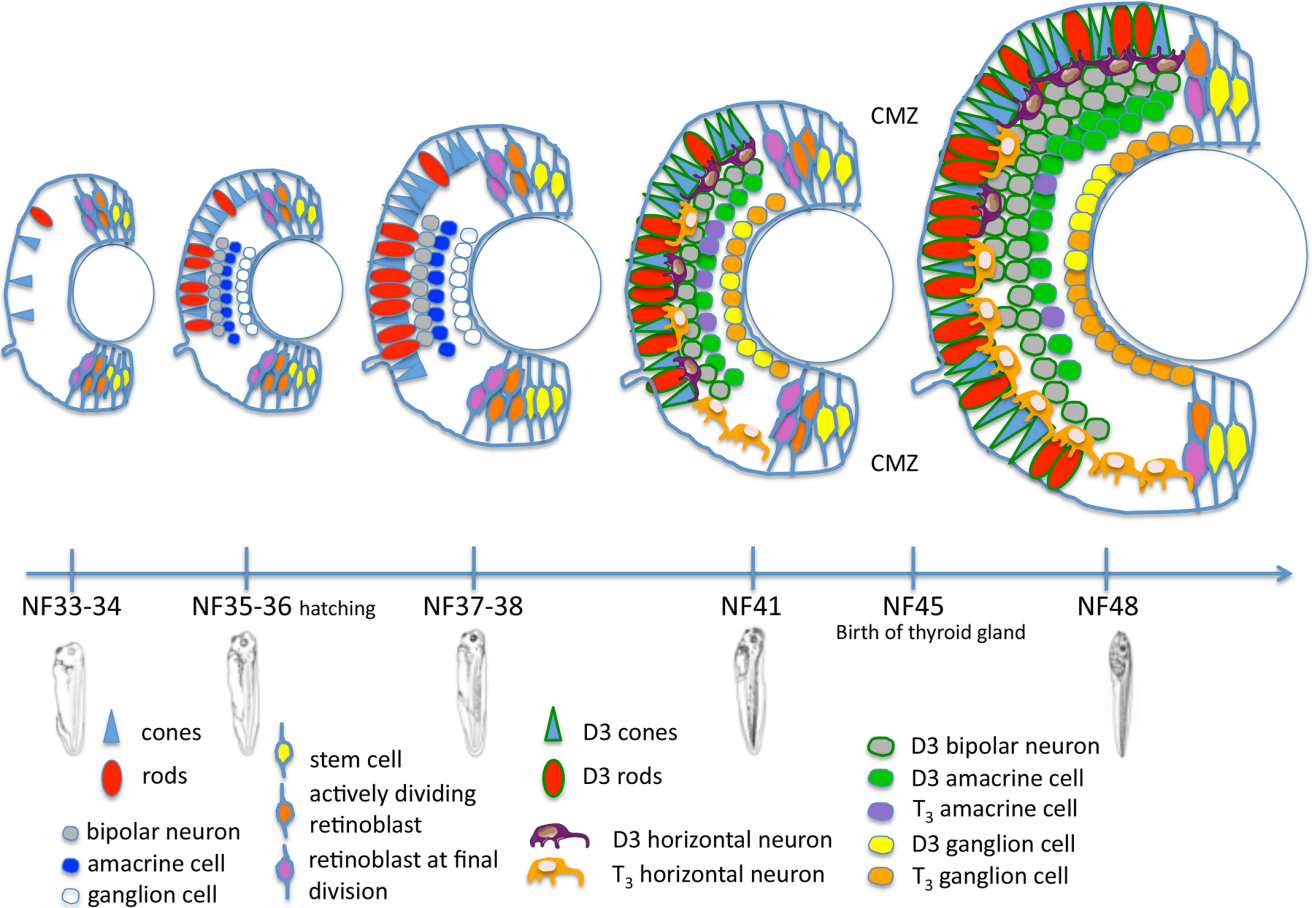
Retinal cell type neurogenesis in pre-metamorphic *Xenopus* from stage NF33 to stage NF48. The schema is based on the following references: Chang WS. and Harris WA. 1998, for photoreceptors determination from stages NF33 to NF41; Bilitou A. and Ohnuma S. 2010, for stem cells and retinoblasts from stages NF33 to NF41; Parker RO. et al. 2010, for violet cone opsin from stages NF35 to NF55; Dullin JP. et al. 2007, for horizontal and amacrine cells for stage NF40-41; Bessodes N. et al. 2017, for amacrine cells for NF41; Álvarez-Hernán G. et al. 2013, for ganglion cells for stage NF35-36; D'Autilia S. et al. 2006, for bipolar neurons for stage NF35-36 (in toto ISH). Results from the current study are used for the different retinal cell-types for stages NF41-48. The key to the different cell types legends is given within the schema.

Our study shows a specific and dynamic spatio-temporal pattern for *dio3* transcripts and *dio3* promoter activity in the retina of pre-metamorphic *Xenopus* tadpoles and post-metamorphic juveniles. The results suggest that the timing of retinal maturation in pre-metamorphic tadpoles is mostly controlled by local, cell-specific D3 activity following modulations between stages NF 41 and NF48. Moreover, D3 activity follows a dorso-ventral gradient at NF48. And the differential-T_3_ responsiveness of retinal cells in our TH sensor model strongly suggests that some photoreceptors and bipolar neurons are more specifically protected from TH-driven maturation at stages NF41 and NF48. Likewise, the same result is observed for amacrine cells at NF48. Our results suggest too that more ventral horizontal neurons and more ganglion cells needed an active TH signaling between NF41 and NF48 (Fig 5).

Therefore, we show that in the pre-metamorphic retina, TH availability is not controlled at the tissue level but at the cellular level by D3, in most of the cell types in each retinal layer. In particular, D3 is expressed in photoreceptors and bipolar neurons at NF41 and NF48 whereas at NF45, only a photoreceptors subset expressed D3. At stage NF48, D3 is expressed specifically in Opsin S cones, rods and bipolar neurons (Fig 5).

The more restricted expression of D3 at stage NF45 could allow the recruitment and the maturation of more cell types of the inner nuclear layer and more pre-existing photoreceptors types. In parallel, maternal stocks of TH decrease at this stage [22]. Then, as the tadpole thyroid gland is formed, T_4_ levels increase at NF46. So, we can speculate that the small subset of photoreceptors expressing D3 at NF45 may correspond to a subtype protected from TH maturation, just before TH levels increase.

At stage NF48, the higher D3 expression observed in most photoreceptors and in cells of the inner nuclear cell layer, could be related to the fact that retina need to be protected from the step increase in TH levels that will initiate pro-metamorphosis at NF54 [31]. Pro-metamorphosis is a step where TH signaling is active and T_3_-dependent tissue remodeling starts [30], illustrated by the onset of green rod differentiation in retina [21].

This result is in accordance with previous studies showing a retinal photoreceptors sensitivity to TH excess during retina development in several species. In vertebrates, TH and TRs are involved in opsin expression and cone development [10, 28, 32, 33, 34, 35]. Furthermore, TH can modulate the M-opsin/S-opsin ratio in the developing mouse retina [29]. Moreover, in trout, excess of TH during smoltification results in a loss of UVS cones [34, 36, 37, 38]. Likewise, another study shows that systemic TH may induce retinal remodelling in juvenile rainbow trout [39].

By analyzing *dio3* and T_3_-target genes transcriptional regulation by T_3_ in the presence or absence of a deiodinase inhibitor (IOP), we show that *dio3* plays a pivotal role in controlling local T_3_ availability in *dio3* expressing cells. No effect of exogenous T_3_ is observed when deiodinases are not inactivated by IOP. The fact that expression of T_3_-responding genes increases when exogenous T_3_ is added in the absence of IOP reflects the fact that these genes must be expressed in cells devoid of *dio3*, such as in the ventral zone.

A final point is that the p*dio*3-GFP reporter tadpoles are a useful tool for following *dio3* expressing cells and for displaying the regulation of TH availability by D3 at the cellular scale. Moreover, the expression level of *dio3* and the reporter gene are reasonably well correlated, despite a certain variability due to the very dynamic D3 expression at specific stages, a fact that could be compounded by the difficulty of obtaining homogeneous batches of tadpoles. However, even if *dio3* is transcriptionally responsive to T_3_, its range of T_3_-responsiveness is far below that of the *TH/bZIP*. As such the *TH/bZIP* reporter remains the best T_3_-sensor available to date in xenopus.

## Conclusions

Our findings show that *dio3* displays a dynamic and cell-specific expression in the pre-metamorphic retina between NF41 and NF48. At stage NF41 and NF48 most cells types express *dio3*, with a particularly high expression in rods and the S cones at NF48 whereas T_3_ signaling is detectable in horizontal neurons and ganglion cells (Fig 5). After metamorphosis, *dio3* expression was much more limited, only being found in amacrine cells and a sub-population of photoreceptors. The results show that *dio3* plays a key role in determining TH availability during retinal development with a precise and cell-type specific timing. We suggest that D3 expression could be necessary to protect photoreceptors and bipolar neurons from out of phase TH signaling and hence that *dio3* expression constrains effects of TH signaling in the retina prior to and during metamorphosis.

## Abbreviations

TH: Thyroid hormone
D3: deiodinase type 3
T4: thyroxine
T3: triiodothyronine
TR: TH receptors
D2: deiodinase type 2
PBS: phosphate buffer saline
NGS: normal goat serum
ISH: in situ hybridization
SSC: saline sodium chloride citrate
GFP: green fluorescent protein
INL: inner nuclear cells layer
ONL: outer nuclear cells layer
OPL: outer plexiform layer
IPL: inner plexiform layer
NF: Nieuwkoop and Faber
TH/bZIP: Thyroid Hormone basic leucine zipper
Chx10: Ceh-10 Homeodomain-Containing Homolog
CMZ: ciliary marginal zone
Opsin S: Opsin Short wavelength
Opsin M: Opsin Medium wavelength
UVS cones: ultraviolet sensitive cones
